# Effect of knowledge of sulfadoxine-pyrimethamine (SP) as prophylaxis for malaria on its uptake for intermittent preventive treatment of malaria in pregnancy (IPTp): Application of inverse probability weighted regression adjustment (IPWRA) technique

**DOI:** 10.1371/journal.pone.0320893

**Published:** 2025-04-15

**Authors:** Charles Natuhamya, Edson Mwebesa, Nazarius Mbona Tumwesigye

**Affiliations:** 1 Makerere University School of Public Health, Kampala, Uganda; 2 Muni University, Arua District, Uganda; Para Federal University, BRAZIL

## Abstract

**Introduction:**

Malaria still remains a global health issue. In response, the World Health Organisation has continuously recommended the use of Sulfadoxine-Pyrimethamine (SP) for Intermittent Preventive Treatment of Malaria in Pregnancy (IPTp) as a malaria preventive measure for the mother and fetus, which has been implemented by the Ugandan government. In collaboration with partners, the government has created awareness of using SP for IPTp (SP-IPTp) among women mainly through media. Studies have investigated the effect of a woman’s education attainment on SP-IPTp. However, the effect of knowledge of SP as prophylaxis for malaria on SP-IPTp has not been studied. Notably, education does not necessarily have an effect on knowledge of SP for malaria prevention, and knowledge of SP as prophylaxis may not result in its significant uptake for IPTp. The purpose of this study, therefore, was to ensure baseline covariate balance and determine the effect of knowledge of SP as preventive chemotherapy on its uptake for IPTp.

**Methods:**

The study utilised the Ugandan Malaria Indicator Survey dataset of 2018–19. Women aged 15–49 years who indicated their uptake status of SP during their last pregnancy formed the sample of this study. The inverse Probability Weighted Regression Adjustment technique was applied to assess the study objective.

**Results:**

The findings revealed a positive and significant effect of knowledge of SP as malaria prophylaxis on its uptake for IPTp (Average Treatment Effect of the Treated or ATET =  0.163; 95% CI =  0.138–0.188).

**Conclusion:**

Ensuring covariate balance while applying IPWRA resulted in more precise estimates of treatment effects. Programmes and policies that create awareness of using SP as malaria prophylaxis may serve as effective interventions towards SP-IPTp in Uganda.

## Introduction

Malaria, caused by the mosquito-transmitted parasite Plasmodium falciparum, is globally a major cause of mortality and morbidity every year [1] and a serious illness [2] that significantly varies at both individual and area levels [3]. The morbidity and mortality caused by malaria are continually increasing [4]. Pregnant women who are uniquely susceptible to malaria infection [5] form part of the disadvantaged groups of individuals besides children, with the highest morbidity and mortality [6], and malaria in pregnancy is associated with a high economic burden on households and the health system [7].

The World Health Organization (WHO) strongly recommended the use of SP-IPTp in areas of moderate to high Plasmodium falciparum malaria transmission like Uganda for all pregnant women irrespective of the number of pregnancies [8] which in response, has been implemented by the Ugandan government [9]. SP is an affordable IPTp option among pregnant women as it is widely available [10] and its benefits for IPTp in malaria-endemic areas of Africa have been well documented [11]. Besides being prophylaxis for malaria, among other benefits of SP-IPTp is that its uptake in higher doses may lead to delivery at term and normal birth weight babies [12]. In Africa, however, average knowledge of SP-IPTp exists among majority of the mothers [13]. In addition, data from 33 countries in the WHO African region showed that only 35% of pregnant women had received the recommended doses of SP-IPTp [8] while in Uganda, still less than half of the women take the recommended dosage [14].

To assess the study objective, IPWRA was applied. Unlike other methods that control for confounding like multivariable regression, IPWRA is justified in case of several confounders or a small number of events. It also retains most individuals in the analysis that otherwise would have been dropped if propensity score matching was applied, resulting in an increased effective sample size [15]. Inverse Probability Weighting (IPW) is useful for adjusting for bias due to confounding or selection in observational studies by weighting [16]. In IPWRA, a logistic regression model is applied to estimate the probability of exposure, and the predicted probability is used for weighting in the subsequent analyses. The inclusion of weights in the analysis achieves covariate balance, a fundamental concept in Randomized Controlled Trials (RCTs) upon which precise causal evidence is based.

Although the use of the technique is rapidly increasing in literature, several published studies have not considered the vital step of assessing the comparability of the treated and control groups in the weighted sample [17]. This study addressed this omission by assessing the balance of baseline covariates between the treated group (women knowledgeable of SP) and the control group (women without knowledge of SP) in the sample weighted by the inverse probability of treatment.

Though some studies have investigated the association of women’s education attainment with SP-IPTp [17,18], they haven’t determined the influence of knowledge of SP on its uptake for IPTp. Since formal education does not necessarily have an effect on knowledge of SP-IPTp [13], it is critical to ascertain the effect of knowledge of SP on SP-IPTp. Hence this study aimed to assess the balance of baseline covariates between women knowledgeable about SP (treated group) and those without such knowledge (control group) using inverse probability of treatment weighting and to determine the effect of SP knowledge on its uptake for IPTp.

## Methods

### Data description and study population

This study utilized secondary data from the Ugandan Malaria Indicator Survey (MIS) of 2018–19, which was the most recent Ugandan MIS at the time of this study. The MIS was based on a two-stage cluster and stratified sampling technique where, at the first sampling stage, a total of 320 clusters were selected with probability proportional to size from the enumeration areas (EAs) covered in the 2014 National Population and Housing Census (NPHC) and 28 households were systematically selected from each EA at the second sampling stage, resulting into a total sample size of 8,878 households [9]. The MIS collected information on vector control interventions such as mosquito nets, indoor residual spraying of insecticides, intermittent preventive treatment of malaria in pregnant women, and malaria knowledge, behaviour, and practices, among others. Women aged 15 to 49 years who were either permanent residents of the selected households or visitors that stayed in the household the night preceding the survey were eligible to be interviewed [9]. The study population consisted of 4,718 women aged 15 to 49 years who indicated their uptake status of SP during their last pregnancy.

### Measurement of variables

#### Dependent variable.

Uptake of SP-IPTp was the dependent variable for this study, and it was measured during the last Ugandan MIS by asking women whether they had taken any dose of SP/Fansidar for malaria prevention during pregnancy. Women who disclosed to have done so were categorized as SP-IPTp users and coded as 1 while their counterparts were categorized otherwise and coded as 0.

#### Independent variables.

The main independent variable was knowledge of SP as prophylaxis for malaria, the treatment variable in this study upon which treatment effects are based. During the last Ugandan MIS, knowledge of SP was measured by asking women whether they were aware of SP/Fansidar as malaria preventive medicine during pregnancy. Responses to this were recorded as No (coded 0) and Yes (coded 1). The outcome model included; education level, number of antenatal care visits, malaria messages, and type of place of residence while the treatment model included variables in the outcome model in addition to wealth index and age group, but excluded the number of antenatal care visits and type of place of residence for correct specification of the model.

To control for variability among variables, the household’s wealth index was re-categorized; ‘poorer’ and ‘poorest’ were grouped as low, ‘richer’ and ‘richest’ as high while ‘middle’ was maintained. For education level, ‘secondary’ and ‘higher’ were combined into secondary or higher while the rest were maintained. For age group, the four highest 5-year age groups were combined into 35 and above, and the rest were maintained. The ‘number of antenatal care visits’ was grouped into less than 4 and 4 or more visits as previously recommended [19].

### Statistical analysis

All statistical analyses were conducted in Stata 15.0 (StataCorp, College Station, TX). Both the treatment and outcome models were first specified before conducting inverse probability-weighted regression adjustments. In the process of model specifications, bivariate analyses were conducted, and later multivariable models fitted on the outcome variables. The backward-step elimination criteria were used while selecting variables for the final outcome and treatment multivariable models.

### Ethics approval

The author was granted permission to use the datasets for the aim of this study. Upon request for the permission, the data were made available for download and use for free. In addition, the 2018–19 Ugandan MIS had received approval from the Uganda National Council for Science and Technology (UNCST), the Ethics Committee of the School of Medicine Research and Ethics Committee (SOMREC) of the Makerere University as well as the institutional review board of the ICF.

### Inverse probability weighted regression adjustment (IPWRA)

RCTs are necessary to establish the highest causal evidence. Through randomization, observed and unobserved participants’ characteristics are typically balanced across groups. But because of some of their limitations like being costly and time-consuming, and ethical limitations, they are rarely carried out. IPWRA is an adjustment technique in observational research that adjusts for baseline characteristics imbalances between treated and non-treated groups [15]. The technique uses propensity score, a conditional probability to a particular treatment vector of baseline individual’s characteristics [20]. Propensity scores can be applied in observational studies in a way analogous to randomized experimental studies [21]. Having carefully considered covariates to be included in the propensity score model, and the appropriate treatment of any extreme weights, IPWRA offers a fairly straightforward analysis approach in observational studies that is analogous to Randomized Controlled Trials (RCTs) [15]. In this study, ATET of knowledge of SP on its use for IPTp was estimated.

ATET was computed as:


$$ATET=E(y_{1}-y_{0}\backslash t=1)$$

Predicted outcomes mean (POM) for treatment level t was calculated as:


$${POM}_{t}=E(y_{t})$$

Each individual’s potential outcomes are $y_{0i}$ and $y_{1i}$ where, $y_{0i}$ is the outcome that would be obtained if i is not knowledgeable of SP for IPTp (not treated), and $y_{1i}$ is the outcome that would be obtained if i is knowledgeable of the same (treated). $y_{0i}$ and $y_{1i}$ are realizations of the random variables $y_{0}$ and $y_{1}.$ The unobservable individual-level treatment effect is $\left(y_{1}-y_{0}\right)$, t denotes a random treatment, $t_{i}$ denotes the treatment received by individual i, t=1 is the treatment level, and t=0 is the control level.

### Balance of baseline covariates

Covariate balance is the degree to which the distribution of covariates is similar across levels of the treatment which is the benefit of randomization in RCTs. While matching, covariate balance was useful for assessing the quality of resulting matches and providing evidence that the estimated treatment effect was close to the true effect.

### Standardized difference

Standardized differences assessed covariate balance in measured baseline covariates between treated and control subjects in the sample that was weighted using inverse probability of treatment [17]. It was expected that baseline covariates in the treatment model would be balanced between the treated and untreated groups [22]. Balance across covariates was numerically checked using standardized differences (Tables 1 and 2) and a standardized difference value greater than 0.1 was considered as a sign of imbalance [23]. The standardized difference, d was computed as [24]:

**Table 1:** Balance for treated and control observations.

Number of observations	Raw	Weighted
Treated observations	2,737	2,370
Control observations	1,981	2,348
Total	4,718	4,718

**Table 2:** Standardized differences and variance ratio values for covariates.

Covariate	Standardized differences		Variance ratio	
	Raw	Weighted	Raw	Weighted
Wealth index	0.54	0.02	1.42	0.99
Education	0.44	0.02	1.15	1.03
Heard messages about malaria	0.27	0.00	1.12	1.00
Age group	0.20	0.03	0.90	0.94

$d=\frac{\left({\hat{p}}_{t}-{\hat{p}}_{c}\right)}{\sqrt{\frac{{\hat{p}}_{t}\left(1-{\hat{p}}_{t}\right)+{\hat{p}}_{c}(1-{\hat{p}}_{c})}{2}}}$

Where ${\hat{p}}_{t}$ and ${\hat{p}}_{c}$ denote the prevalence of dichotomous variables in the treated (women knowledgeable about SP) and control (women not knowledgeable about SP) group, respectively.

### Variance ratio

This is the ratio of the variance of a covariate in one treatment group to the variance of the same covariate in the other group. Covariate balance was also demonstrated by the variance ratio where a good matching procedure reduced bias by increasing the balance and decreasing the variance [25]. Hence, a variance ratio value close to 1 was considered to demonstrate a good matching [22], indicating that the variances of the groups were similar. The variance ratio, vr was computed as:


$$vr=\frac{{\hat{v}}_{t}}{{\hat{v}}_{c}}$$

Where ${\hat{v}}_{t}$ and ${\hat{v}}_{c}$ represent the variance of dichotomous variables in the treated and control groups, respectively.

The balance of covariates was further graphically presented using Kernel density plots with Epanechnikov Kernel function. The over-identification test was conducted as the overall test for balance based on the hypothesis that; the covariates were balanced. Treatment effects were therefore considered accurate if the null hypothesis of the over-identification test was not rejected.

## Results

This section presents selected characteristics of women aged 15 to 49 years and the treatment effects of knowledge of SP as malaria preventive medicine on its uptake for IPTp.

### Characteristics of the study population of women aged 15 to 49 years

Most women were young; aged between 15 and 24 years 1,577 (33.5%), had attained utmost primary level of education 2,673 (56.7%), had visited health facilities for antenatal care at least 4 times 2,757 (58.4%), had not seen/heard malaria messages 2,804 (59.4%) and were not or unsure of being pregnant by the time of the survey 4,246 (90.0%). Most of these women dwelled in households with low wealth index (poor and poorer) 2,643 (56.0%), resided in rural areas 3,401 (72.1%), and were from the northern region 1,663 (35.3%). The rest of the results are presented in Table 3.

**Table 3:** Distribution of the study population of women aged 15 to 49 years by selected background characteristics from Ugandan MIS of 2018–19.

Characteristic	Category	Count	Percent
**Age**	15–24	1,577	33.5
	25–29	1,179	25.0
	30–34	962	20.4
	35 and above	1,000	21.2
**Education level**	None	938	19.9
	Primary	2,673	56.7
	Secondary and higher	1,107	23.5
**ANC visits**	Less than 4	1,961	41.6
	4 or more	2,757	58.4
**Saw/heard any malaria messages**	No	2,804	59.4
	Yes	1,914	40.6
**Currently pregnant**	No or unsure	4,246	90.0
	Yes	472	10.0
**Wealth index**	Low	2,643	56.0
	Middle	756	16.0
	High	1,319	28.0
**Type of place of residence**	Urban	932	19.8
	Rural	3,401	72.1
	Refugee	385	8.2
**Region**	Central	736	15.6
	Eastern	1,020	21.6
	Northern	1,663	35.3
	Western	1,299	27.5

### Covariate balance results

Table 1 indicates a significant balance between the treated and control observations after weighting as opposed to before weighting. The weighted standardized differences values across the covariates were less than 0.1 indicating balance in the covariates after matching (see Table 2). After weighting, the variance ratio values of all covariates were approximately 1, which is also an indication of covariate balance (Table 2).

**Fig 1:**
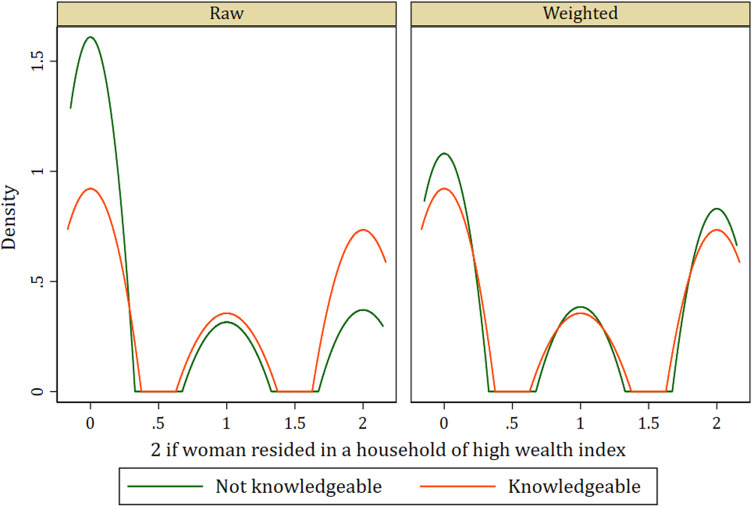
Kernel density plot showing covariate balance for household’s wealth index.

**Fig 2:**
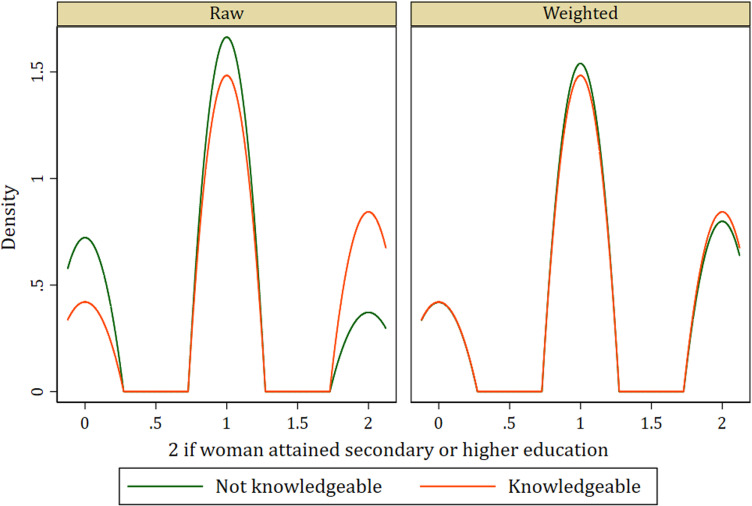
Kernel density plot showing covariate balance for education level.

**Fig 3:**
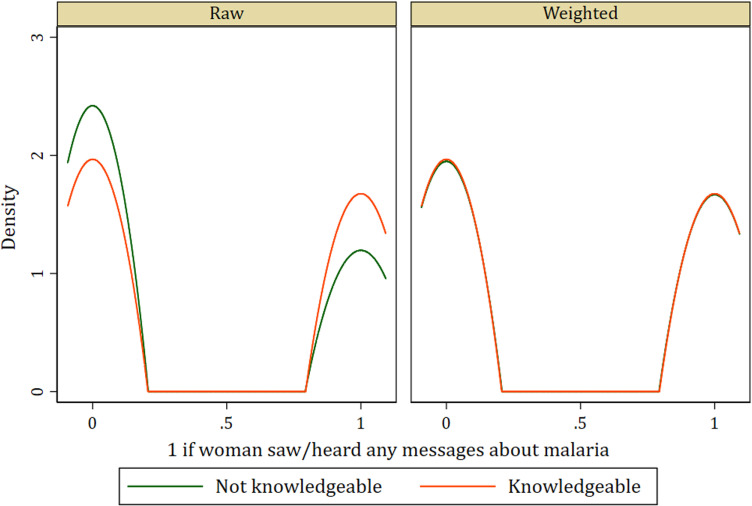
Kernel density plot showing covariate balance for malaria messages.

**Fig 4:**
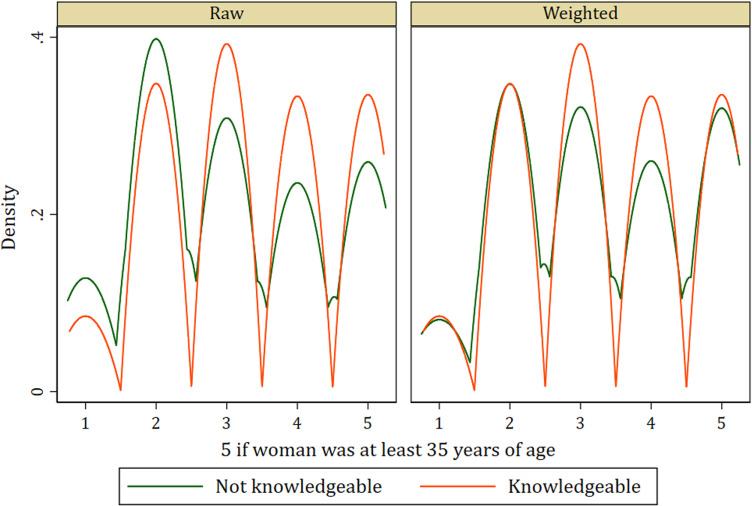
Kernel density plot showing covariate balance for age.

In addition to the standardized differences and variance ratio, the Kernel density graphs evaluating covariate balance in the treatment model among women who were knowledgeable about SP as malaria preventive medicine and those who were not knowledgeable of the same show similar distributions after weighting. This indicates a balance among covariates (see Figs 1–4).

### Treatment effects of knowledge of SP as preventive chemotherapy on its uptake for IPTp

Results from the treatment model show that the probability of using SP for IPTp was 16% (ATET =  0.163; 95% CI =  0.138–0.188) higher among women who were knowledgeable about SP as malaria prophylaxis compared to 79% (POM =  0.794; 95% CI =  0.770–0.817) if none of these mothers were knowledgeable about the same. This indicates women’s awareness of SP as prophylaxis for malaria increases the likelihood of using it for IPTp by 16% compared to when women are unaware of it.

Further, after converting ATET as a percentage, results in Table 4 indicate that the probability of using SP for IPTp increased by an estimated 21% (ATET =  0.205; 955 CI =  0.168–0.242) when every mother was knowledgeable about SP as malaria prophylaxis relative to the case when no mothers was knowledgeable of the same.

**Table 4:** Estimation of ATET of Knowledge of SP on its use for IPTp (weighted versus unweighted data).

	Unweighted/unbalanced		Weighted/balanced	
	Coefficient (95% CI)	SE	Coefficient (95% CI)	SE
**ATET**				
Knowledgeable about SP (yes versus no)	0.147 (0.129, 0.166)*	0.010	0.163 (0.138, 0.188)*	0.013
**POM**				
Knowledgeable about SP (no)	0.809 (0.791, 0.826)*	0.009	0.794 (0.770, 0.817)*	0.012
**Proportion of ATET (%)**	0.182 (0.155, 0.209)*	0.014	0.205 (0.168, 0.242)*	0.019

Over-identification test for covariate balance: Chi2(5) = 6.6478; p = 0.2482.

ATET, average treatment effect on the treated; CI, confidence interval; POM, predicted outcomes mean; SE, standard error; Chi2, chi-square; * p <  0.001.

Results from the over-identification test in Table 4 indicate that the null hypothesis was not rejected. This showed that the treatment model balanced the covariates, proving accuracy of the treatment effects.

## Discussion

The study ensured covariate balance while applying IPWRA to assess the effect of knowledge of SP on SP-IPTp among pregnant women. The study utilised data from a nationally representative sample to make inferences about the Ugandan population.

Results from this study indicate relatively small standard errors and different parameter estimates in a model devoid of weighting (with unbalanced covariates) compared to one with balanced covariates. These findings are consistent with previous findings where covariate imbalance resulted in parameter bias and small standard error bias [26].

Even in RCTs, it is difficult to adjust for all prognostic covariates at the design level since some of them may be unknown or unmeasurable [27]. This study adds to the existing literature by demonstrating the importance of adjusting for covariate balance during analysis. Previous findings [28] indicate that properly adjusting for covariate imbalance during analysis annuls the undesirable effect of imbalance. This shows that even though observational studies suffer from drawbacks that experimental studies address during the design stage, ensuring covariate balance contributes to unbiased estimates in observational studies which is useful for estimating results from studies based on experimental designs. Hence, leveraging the baseline information to achieve balanced covariates during analysis [29] can significantly increase the study power [30].

This study found that women’s awareness of SP as prophylaxis for malaria increases the likelihood of using it for IPTp by 16% compared to when women are unaware of it. Although a previous study demonstrated that knowledge about malaria preventive measures did not essentially lead to enhanced malaria prevention practices [31], a recent study found that enhanced women’s knowledge of such measures was significantly associated with their use [32]. Relatedly, the likelihood of using antimalarial drugs by pregnant women was lower among those who did not receive malaria knowledge on the radio compared with those who did [33].

Since mothers’ level of formal education is not necessarily associated with knowledge and use of SP-IPTp [13], targeted educational programs to enhance attitudes and practices regarding malaria control [31] may serve as a better alternative because it was recently found out that although mothers had sufficient knowledge on malaria preventive methods, most of them were adamant in using them [34]. In addition, sensitization targeted towards the use of known preventive measures should be intensified [34] as well as rigorous behavioural communication intervention to improve the knowledge of malaria regarding malaria prevention measures [35] through proper community channels [36], to bridge the existing knowledge gap.

Other studies indicated enhanced use of malaria prevention methods as a result of malaria knowledge through messaging for example, messages to the public about insecticide-treated nets (ITNs) were very useful in increasing the use of the mosquito nets [37], mobile phone short message service (SMS) was effective in malaria control [38], strengthening topic-specific malaria messages was vital for effective malaria communication [39], and utilization of the two peak hours for broadcasting malaria radio interventions was helpful in practicing malaria prevention methods [40].

However, it is worth noting that IPTW does not control for unmeasured or unknown confounding. Hence in case of unmeasured confounding, this may still impact the validity of the effects of knowledge of SP as preventive chemotherapy on uptake of IPTp in this study.

The strength of this study was the national representativeness of the survey data. The study limitations included; the possibility of recall bias since information was purely based on self-report by the survey respondents. However, this was minimal since most responses were only required about events from the most recent past. Also, some variables deemed important may not have been collected however, the variables available in these data sufficiently addressed the study objective.

## Conclusions and recommendations

Ensuring covariate balance while applying IPWRA resulted in unbiased estimates of treatment effects. Hence malaria researchers can use the technique to estimate causal parameters in settings where RCTs are not feasible. The results from this study indicate a significant and positive effect of knowledge of SP as preventive chemotherapy on its uptake for Intermittent Preventive Treatment of Malaria in Pregnancy among mothers in Uganda. Programmes and policies that create awareness of the use of SP as malaria preventive medicine may serve as effective interventions towards its use in Uganda for malaria prevention and control. Identifying the most effective channels for disseminating knowledge of SP may contribute to closing the knowledge gap among pregnant women.

## Abbreviations

ATET: average treatment effect on the treated
EA: enumeration area
IPTp: intermittent preventive treatment of malaria in pregnancy
IPW: inverse probability weighting
IPWRA: inverse probability weighted regression adjustment
ITN: insecticide-treated net
MIS: malaria indicator survey
NPHC: National Population and Housing Census
POM: predicted outcomes mean
RCT: randomized controlled trial
SMS: short message service
SP: sulfadoxine-pyrimethamine
SP-IPTp: sulfadoxine-pyrimethamine for intermittent preventive treatment of malaria in pregnancy
TX: Texas
WHO: World Health Organization.
